# Prevalence of depression and its association with quality of life among guardians of hospitalized psychiatric patients during the COVID-19 pandemic: a network perspective

**DOI:** 10.3389/fpsyt.2023.1139742

**Published:** 2023-05-12

**Authors:** Yan-Jie Zhao, Ling Zhang, Yuan Feng, Sha Sha, Mei Ieng Lam, Yue-Ying Wang, Jia-Xin Li, Zhaohui Su, Teris Cheung, Gabor S. Ungvari, Todd Jackson, Feng-Rong An, Yu-Tao Xiang

**Affiliations:** ^1^Beijing Key Laboratory of Mental Disorders, National Clinical Research Center for Mental Disorders & National Center for Mental Disorders, Beijing Anding Hospital and the Advanced Innovation Center for Human Brain Protection, Capital Medical University, Beijing, China; ^2^Unit of Psychiatry, Department of Public Health and Medicinal Administration, Institute of Translational Medicine, Faculty of Health Sciences, University of Macau, Taipa, Macao SAR, China; ^3^Kiang Wu Nursing College of Macau, Macau, Macao SAR, China; ^4^Centre for Cognitive and Brain Sciences, University of Macau, Taipa, Macao SAR, China; ^5^School of Public Health, Southeast University, Nanjing, China; ^6^School of Nursing, Hong Kong Polytechnic University, Hong Kong, Hong Kong SAR, China; ^7^Section of Psychiatry, University of Notre Dame Australia, Fremantle, WA, Australia; ^8^Division of Psychiatry, School of Medicine, University of Western Australia, Perth, WA, Australia; ^9^Department of Psychology, University of Macau, Taipa, Macao SAR, China

**Keywords:** depression, quality of life, guardians, hospitalized psychiatric patients, network

## Abstract

**Background:**

The COVID-19 pandemic has greatly affected treatment-seeking behaviors of psychiatric patients and their guardians. Barriers to access of mental health services may contribute to adverse mental health consequences, not only for psychiatric patients, but also for their guardians. This study explored the prevalence of depression and its association with quality of life among guardians of hospitalized psychiatric patients during the COVID-19 pandemic.

**Methods:**

This multi-center, cross-sectional study was conducted in China. Symptoms of depression and anxiety, fatigue level and quality of life (QOL) of guardians were measured with validated Chinese versions of the Patient Health Questionnaire – 9 (PHQ-9), Generalized Anxiety Disorder Scale – 7 (GAD-7), fatigue numeric rating scale (FNRS), and the first two items of the World Health Organization Quality of Life Questionnaire - brief version (WHOQOL-BREF), respectively. Independent correlates of depression were evaluated using multiple logistic regression analysis. Analysis of covariance (ANCOVA) was used to compare global QOL of depressed versus non-depressed guardians. The network structure of depressive symptoms among guardians was constructed using an extended Bayesian Information Criterion (EBIC) model.

**Results:**

The prevalence of depression among guardians of hospitalized psychiatric patients was 32.4% (95% *CI*: 29.7–35.2%). GAD-7 total scores (*OR* = 1.9, 95% *CI*: 1.8–2.1) and fatigue (*OR* = 1.2, 95% *CI*: 1.1–1.4) were positively correlated with depression among guardians. After controlling for significant correlates of depression, depressed guardians had lower QOL than non-depressed peers did [*F*_(1, 1,101)_ = 29.24, *p* < 0.001]. “*Loss of energy*” (item 4 of the PHQ-9), “*concentration difficulties*” (item 7 of the PHQ-9) and “*sad mood*” (item 2 of the PHQ-9) were the most central symptoms in the network model of depression for guardians.

**Conclusion:**

About one third of guardians of hospitalized psychiatric patients reported depression during the COVID-19 pandemic. Poorer QOL was related to having depression in this sample. In light of their emergence as key central symptoms, “*loss of energy*,” “*concentration problems*,” and “*sad mood*” are potentially useful targets for mental health services designed to support caregivers of psychiatric patients.

## 1. Introduction

The coronavirus disease 2019 (COVID-19) was first reported in Wuhan, Hubei province of China at the end of 2019 and subsequently emerged in other parts of the world (1, 2). Notwithstanding its negative impact on the health and security of humanity, the COVID-19 pandemic has also had pronounced effects on mental health status and quality of life (QOL) in various populations (3–5).

In times of pandemics, people with mental disorders are more vulnerable to respiratory tract infections (6). Possible correlates of this risk include higher smoking rates, poor personal hygiene and negligence of infection risks due to cognitive impairment as well as crowded living conditions and lack of personal protective equipment (PPE) in psychiatric wards (6–9). As a result of such factors, it is reasonable to hypothesize that hospitalized psychiatric patients are more susceptible to COVID-19. Indeed, this contention was supported early in the pandemic when at least 50 hospitalized psychiatric patients and 30 mental health professionals in a major psychiatric hospital in Wuhan, China were diagnosed with COVID-19 in early 2020 (7, 10). Additionally, a study based on electronic health records in the United States found that patients with psychiatric disorders had a higher risk for COVID-19 infection than those without psychiatric disorders (adjusted OR = 7.64 for depression; adjusted OR = 7.34 for schizophrenia) (11). Two other studies in Spain found that 45% of COVID-19 inpatients had history of psychiatric disorders and 37% of COVID-19 inpatients had medical conditions (12, 13), supporting the view that psychiatric patients are more prone to COVID-19.

In order to minimize infection risk, policies to prevent unnecessary visits and social contacts in hospitals and psychiatric wards were implemented and multiple clinical services were curtailed during early stages of the COVID-19 pandemic (8, 14–16). Psychiatric patients and their guardians have been confronted with numerous barriers in accessing mental health services during the COVID-19 pandemic including difficulties in visiting psychiatrists, reduced access to psychotropic medications and hospital admissions, and problems with evaluating degree of compliance with recommended treatment protocols (8, 16, 17). All of these barriers to optimal psychiatric care could increase risk for depression and reduced quality of life (QOL), not only among patients but also among their guardians. Previous studies have revealed that guardians of adolescents with Type 1 diabetes and isolated COVID-19 patients suffered from higher levels of depression, anxiety and pandemic-related worry compared to adults who did not have family members who were ill during the COVID-19 pandemic (18–20); these findings underscore the importance of considering the mental health status of guardians who must care for psychiatric patients and undertake relevant obligations during the COVID-19 pandemic.

To date, the impact of the COVID-19 pandemic on the mental health status of psychiatric patients has been widely investigated (21–24). In contrast, there has been a paucity of research on the mental health status and QOL of guardians of the hospitalized psychiatric patients. Documenting the prevalence of depression as well as its correlates and association with QOL among guardians of psychiatric patients during the pandemic is important for ensuring close support systems of patients are maintained and distressed caregivers also have access to interventions that reduce their own suffering.

Traditionally, epidemiological research on depression has adopted a latent factor approach (25) in which depression is regarded as an unobservable, latent factor and depressive symptoms are observable manifestations or indicators of depression (26). Key assumptions underlying the latent factor approach are that all symptoms are present or dependent upon one another and equally important in their contributions to overall depression levels (25, 26). However, symptoms such as anhedonia, hopelessness and reduced energy often have robust associations with each other even when diagnostic criteria for MDD are not fulfilled (27, 28). Such data highlight how traditional latent factor approaches cannot elucidate inter-relationships between different depressive symptoms although individual symptoms may play an important role in the onset and maintenance of depression (29, 30). As an alternative to the traditional perspective, a network approach may provide more understanding of how depressive symptoms are interconnected, particular symptoms that are most influential for the syndrome within particular populations (31–33).

Based on the preceding overview, the initial aim of this study was to document the prevalence of depression, its correlates, and its association with QOL among guardians of hospitalized psychiatric patients during the COVID-19 pandemic. In addition, we used network analysis to generate a network model of inter-relations between specific depressive symptoms within this understudied group.

## 2. Methods

### 2.1. Study setting and participants

This multi-center, cross-sectional study was conducted between May 24, 2020 and January 18, 2021 in seven tertiary psychiatric hospitals and psychiatric units of general hospitals in China. To avoid COVID-19 infection risk, data were collected using the WeChat-based QuestionnaireStar application as recommended in previous studies (34, 35). Guardians needed to declare their health status using WeChat during the COVID-19 pandemic when they entered participating hospitals. Therefore, all guardians were presumed to be WeChat users. Guardians who visited hospitalized patients during the study period were consecutively invited to participate. Inclusion criteria were as follows: (1) age 18 years or older; (2) ability to read Chinese and understand the purpose and contents of the assessments; (3) status as a guardian (e.g., spouse, child, parent, other kin or friend) of a hospitalized psychiatric patient in participating hospitals; (4) provision of online electronic informed consent. Guardians with a psychiatric history or current psychiatric disorders were excluded from this study since this was a possible confounding factor to estimating depression prevalence for guardians as a population distinct from psychiatric patients. The study protocol was centrally approved by the research ethics committee of Beijing Anding Hospital, Capital Medical University and other participating hospitals.

The data collection form was designed using the QuestionnaireStar application. A Quick Response (QR) code linked to the informed consent and data collection form was scanned by the participants with their smart phones. Those who met eligibility criteria completed the assessment in participating hospitals on a voluntary, anonymous basis.

### 2.2. Data collection and assessment tools

Socio-demographic data assessed included age, gender, marital status, employment status, education level, urban versus rural residence, presence of chronic physical diseases, perceived financial status, frequency of social media use during the COVID-19 pandemic, and experience of difficulty in visiting mental health services during the pandemic.

Severity of depressive symptoms was assessed using the validated Chinese version of the Patient Health Questionnaire – 9 (PHQ-9). The PHQ-9 consists of nine items, each rated on a frequency scale from 0 (not at all) to 3 (almost every day) (36, 37). Higher PHQ-9 scores represent more severe depression (38). The reliability and validity of the PHQ-9 are satisfactory in Chinese populations (39, 40). Participants were regarded “having clinically relevant depression” (having depression hereafter) if their total PHQ-9 score was ≥5 (38).

Severity of anxiety symptoms was assessed using the Chinese version of the Generalized Anxiety Disorder Scale – 7 (GAD-7). The GAD-7 consists of seven self-report items, each of which is rated on a frequency scale from 0 (not at all) to 3 (almost every day) (41); higher GAD-7 scores reflect more severe anxiety symptoms. The GAD-7 Chinese version has been validated in Chinese populations (42, 43). Level of fatigue was evaluated using a single-item fatigue numeric rating scale (FNRS) (44). FNRS scores range from 0 (no fatigue) to 10 (extreme fatigue).

Global quality of life (QOL) was assessed with the first two items of the World Health Organization Quality of Life Questionnaire - brief version (WHOQOL-BREF) (45, 46). These items queried overall quality of life and general health status from 1 (extremely unsatisfied) to 5 (extremely satisfied) (47). This two-item QOL index has been validated and used widely in Chinese samples (48).

### 2.3. Data analyses

All data analyses were conducted using Statistical Analysis System (SAS) OnDemand for Academics (SAS Institute Inc., Cary, NC, United States) and R version 4.2.1 (49). Sociodemographic and emotional status differences between depressed versus non-depressed guardian subgroups were assessed using independent two-sample *t-*tests, Wilcoxon rank sum tests, and chi-square tests, as appropriate. Analysis of covariance (ANCOVA) was used to compare global QOL score differences between depressed versus non-depressed guardians after first controlling the impact of other measures on which there were subgroup differences in univariable analyses (i.e., covariates). Independent predictors of depression levels were evaluated using a multiple logistic regression analysis; depression was the dependent variable, and significant univariate correlates of depression subgroup status were predictors in the analysis. Age and sex are generally associated with mental health status and QOL in many populations (50); therefore, they were included as potential predictors in the multiple logistic regression model, even though neither had significant associations with depression in univariate analyses. In addition, independent predictors of depression were explored separately for first-degree relatives (spouse, children, and parents). Two-sided *p-*values lower than 0.05 were considered to be statistically significant.

To capture the full spectrum of depression severity and increase external validity, the network structure of depressive symptoms was constructed for all guardians of hospitalized psychiatric patients rather than only the depressed guardians, as recommended by previous studies (51, 52). An extended Bayesian Information Criterion (EBIC) model graphical least absolute shrinkage and selection operator (gLASSO) network model was adopted in this study. In the network structure, each individual symptom was a “node,” and connections between symptoms were “edges.” The centrality of each symptom was measured using strength, defined as the sum of the absolute weights of the edges connecting a certain node to all the other nodes. The size of a node represented the strength of a particular symptom. The thickness of each edge represented the strength of the association between two nodes. The color of an edge reflected the direction of the association with green edges indicating positive associations and red edges indicating negative associations between nodes.

Network stability was examined *via* the correlation stability coefficient (*CS-C*) using a case-dropping 1,000-time bootstrap method (53, 54). Preferably, a *CS-C* exceeds 0.5, with a minimum value requirement of 0.25 (55).

To examine the impact of anxiety symptoms and fatigue on the observed network structure of depressive symptoms, the network model of depression was re-estimated after adjusting for anxiety symptoms and fatigue. A flow network was applied to investigate relationships between individual depressive symptoms and QOL. R packages used in this study were *networktools version 1.2.3* (56), *bootnet version 1.4.3* (55), *qgraph version 1.6.5* (57), *NetworkComparisonTest version 2.2.1* (58, 59), and *mgm version 1.2–10* (60).

## 3. Results

### 3.1. Sociodemographic and clinical characteristics of guardian sample

In total, 1,163 guardians of hospitalized psychiatric patients were invited to participate in this study; of these, 1,101 (94.7%) agreed to participate, fulfilled the eligibility criteria, and completed the assessment. Table 1 presents demographics and clinical characteristics of final guardian sample. The prevalence of depression among guardians of hospitalized psychiatric patients was 32.4% (95% *CI*: 29.7–35.2%).

**Table 1:** Demographic and clinical characteristics of the study sample (*N* = 1,101).

Variables	Total (*N* = 1,101)		No depression (*N* = 744)		Depression (*N* = 357)		Univariable analysis		
	*n*	%	*n*	%	*n*	%	*χ* ^2^	*df*	*p*
Male	442	40.1	293	39.4	149	41.7	0.6	1	0.45
Married	903	82.0	617	82.9	286	80.1	1.3	1	0.25
Employed	894	81.2	614	82.5	280	78.4	2.7	1	0.10
Senior secondary school and above	677	61.5	448	60.2	229	64.1	1.6	1	0.21
Living in rural areas	468	42.5	318	42.7	150	42.0	0.1	1	0.82
Presence of major physical diseases	52	4.7	29	3.9	23	6.4	3.5	1	0.06
Perceived financial status							14.4	2	**<0.001**
Poor	234	21.3	144	19.4	90	25.2			
Fair	760	69.0	512	68.8	248	69.5			
Good	107	9.7	88	11.8	19	5.3			
Frequency of social media use during the pandemic							7.4	2	**0.024**
No or minimal	85	7.7	58	7.8	27	7.6			
Sometimes	356	32.3	221	29.7	135	37.8			
Often	660	59.9	465	62.5	195	54.6			
Difficulty visiting mental health service during the pandemic	327	29.7	175	23.5	152	42.6	42.0	1	**<0.001**
	Mean	SD	Mean	SD	Mean	SD	t/Z	df	*p*
Age (years)	43.1	11.6	43.3	11.4	42.5	12.1	1.05	1,099	0.29
GAD-7 total	3.0	4.6	0.8	1.7	7.8	5.2	24.6	—	**<0.001**
Fatigue	3.1	2.5	2.3	2.2	4.7	2.3	15.3	—	**<0.001**
Global QOL	6.6	1.7	7.1	1.4	5.4	1.5	17.8	1,099	**<0.001**
Patient information									
	*n*	%	*n*	%	*n*	%	$\chi^{2}$	*df*	*p*
Principal psychiatric diagnosis of patient							6.4	3	0.09
Major depressive disorder	400	36.3	262	35.2	138	38.7			
Bipolar disorder	162	14.7	102	13.7	60	16.8			
Schizophrenia	222	20.2	149	20	73	20.4			
Other	317	28.8	231	31	86	24.1			
Good medication compliance during the pandemic	775	70.4	543	73	232	65	7.4	1	**0.007**

Bolded values: <0.05; df, degree of freedom; GAD-7, Generalized Anxiety Disorder – 7 items; SD, standard deviation.

Compared to their non-depressed peers, guardians with depression were more likely to report poorer financial status, difficulty visiting a mental health service during the pandemic, increased fatigue, and elevations in anxiety symptoms. Depressed guardians were also significantly less likely to report that their loved ones showed good compliance with medication during the pandemic and had a lower mean overall QOL level (all *p-*values<0.05; see Table 1). In contrast, depressed versus non-depressed guardian subgroups did not differ on any demographic measure.

### 3.2. Global QOL differences between depressed versus non-depressed guardians

After adjusting for other significant correlates of depression status, guardians with depression continued to have a significantly lower mean QOL level than non-depressed guardians had [*F*_(1, 1,101)_ = 29.24, *p* < 0.001].

### 3.3. Predictors of depression among guardians of hospitalized psychiatric patients

The multiple logistic regression analysis indicated higher total GAD-7 scores (*OR* = 1.9, 95% *CI*: 1.8–2.1) and fatigue scores (*OR* = 1.2, 95% *CI*: 1.1–1.4) were the only unique, statistically significant predictors of elevated depression levels within the guardian sample (see Table 2). In a subgroup analysis of first-degree relative guardians, findings were similar to those for the whole sample (see Supplementary Table S1).

**Table 2:** Independent correlates of depression among guardians of hospitalized psychiatric patients during the COVID-19 pandemic (*N* = 1,101).

Variables	Multiple logistic regression analysis		
	*p*	OR	95% CI
Age (years)	0.77	1.0	0.98–1.02
Female	0.56	0.9	0.6–1.4
Presence of major physical diseases	0.75	0.8	0.3–2.4
Perceived financial status (poor vs. fair/good)	0.43	1.2	0.7–2.0
Frequency of social media use (often vs. no or minimal/sometimes)	0.76	0.9	0.6–1.5
Difficulty in visiting mental health service during the pandemic	0.21	1.3	0.9–2.1
GAD-7 total	**<0.001**	1.9	1.8–2.1
Fatigue	**<0.001**	1.2	1.1–1.4
Principal psychiatric diagnosis			
Major depressive disorder	0.81	1.1	0.6–1.8
Bipolar disorder	0.08	1.8	0.9–3.4
Schizophrenia	0.23	1.4	0.8–2.6
Others	—	—	—
Medication compliance during the pandemic (poor vs. good)	0.64	1.1	0.7–1.8

Bolded values: <0.05; CI, confidence interval; GAD-7, Generalized Anxiety Disorder – 7 items; OR, odds ratio.

### 3.4. Network analysis

**Figure 1:**
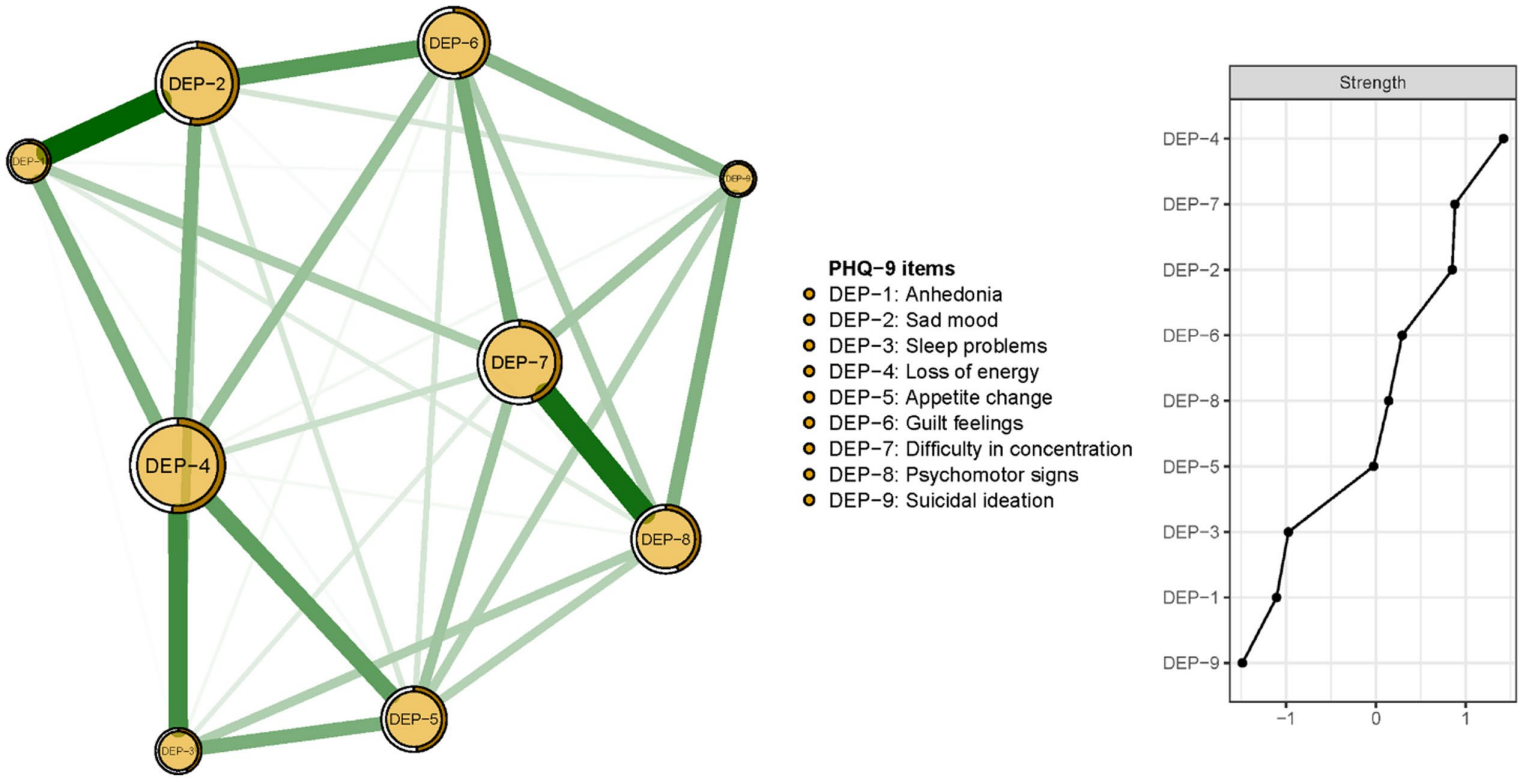
Network structure and strength of the depressive symptoms among guardians of hospitalized psychiatric patients (*N* = 1,101).

**Figure 2:**
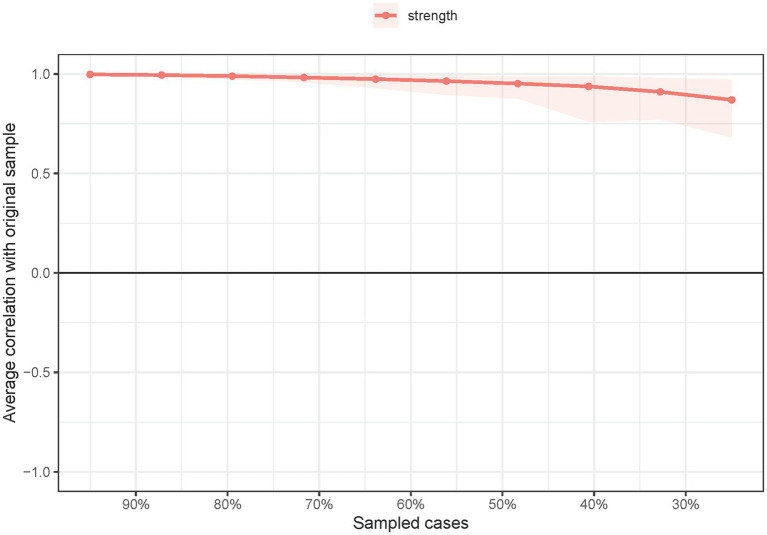
Network stability of depressive symptoms among guardians of hospitalized psychiatric patients (*N* = 1,101).

The network structure of depressive symptoms, as estimated with the EBIC glasso model, is shown in Figure 1. PHQ-9 items 4 (DEP-4, loss of energy), 7 (DEP-7, concentration difficulties), and 2 (DEP-2, sad mood) had the highest strengths in the network model of depressive symptoms. Exact centrality strength values are shown in Supplementary Table S2. The *CS-C* for network model strength was 0.75, indicating that centrality strength values in the network remained stable after dropping 75% of the sample (Figure 2).

**Figure 3:**
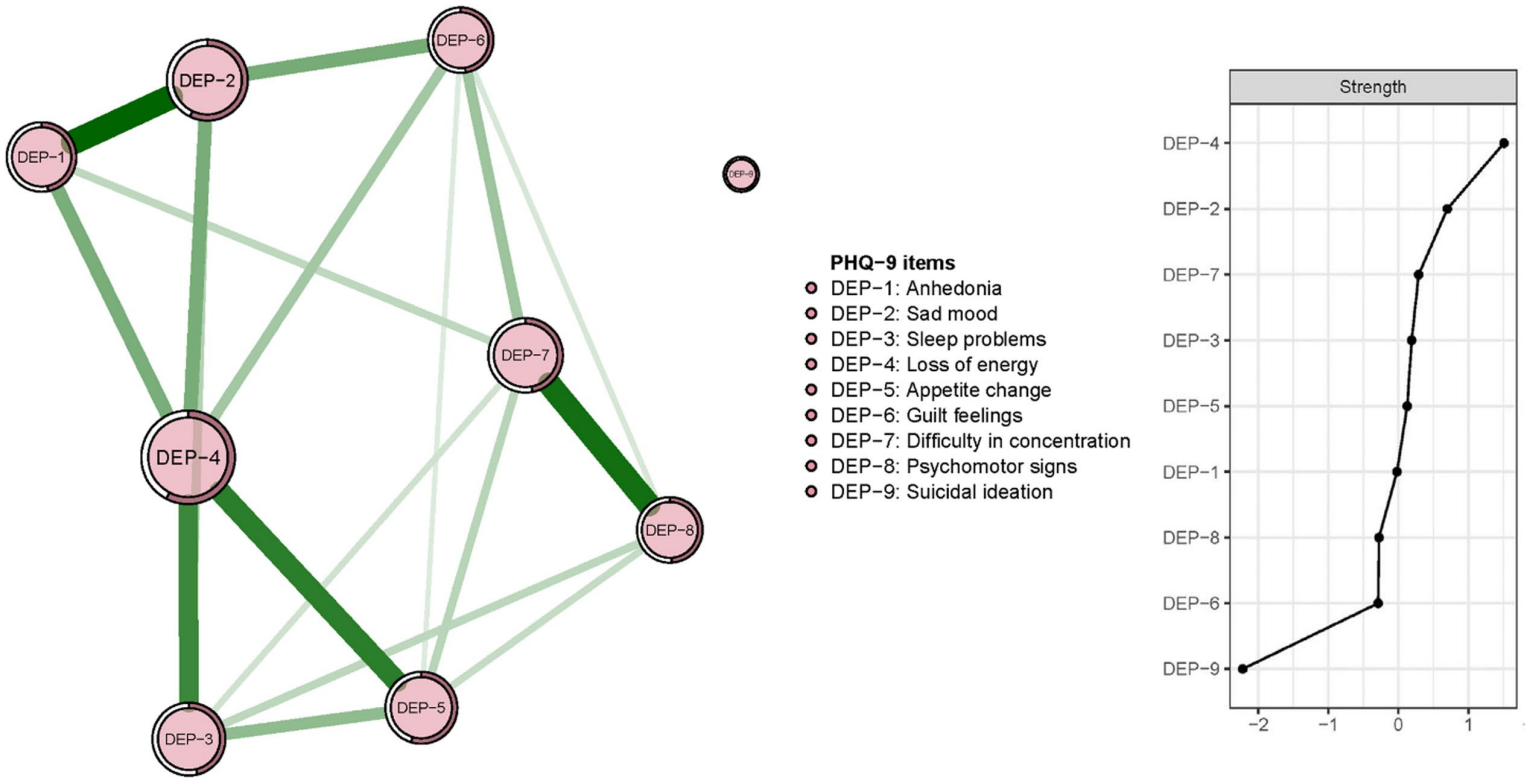
Network structure and strength of depressive symptoms among guardians of hospitalized psychiatric patients after adjusting for anxiety symptoms and fatigue (*N* = 1,101).

**Figure 4:**
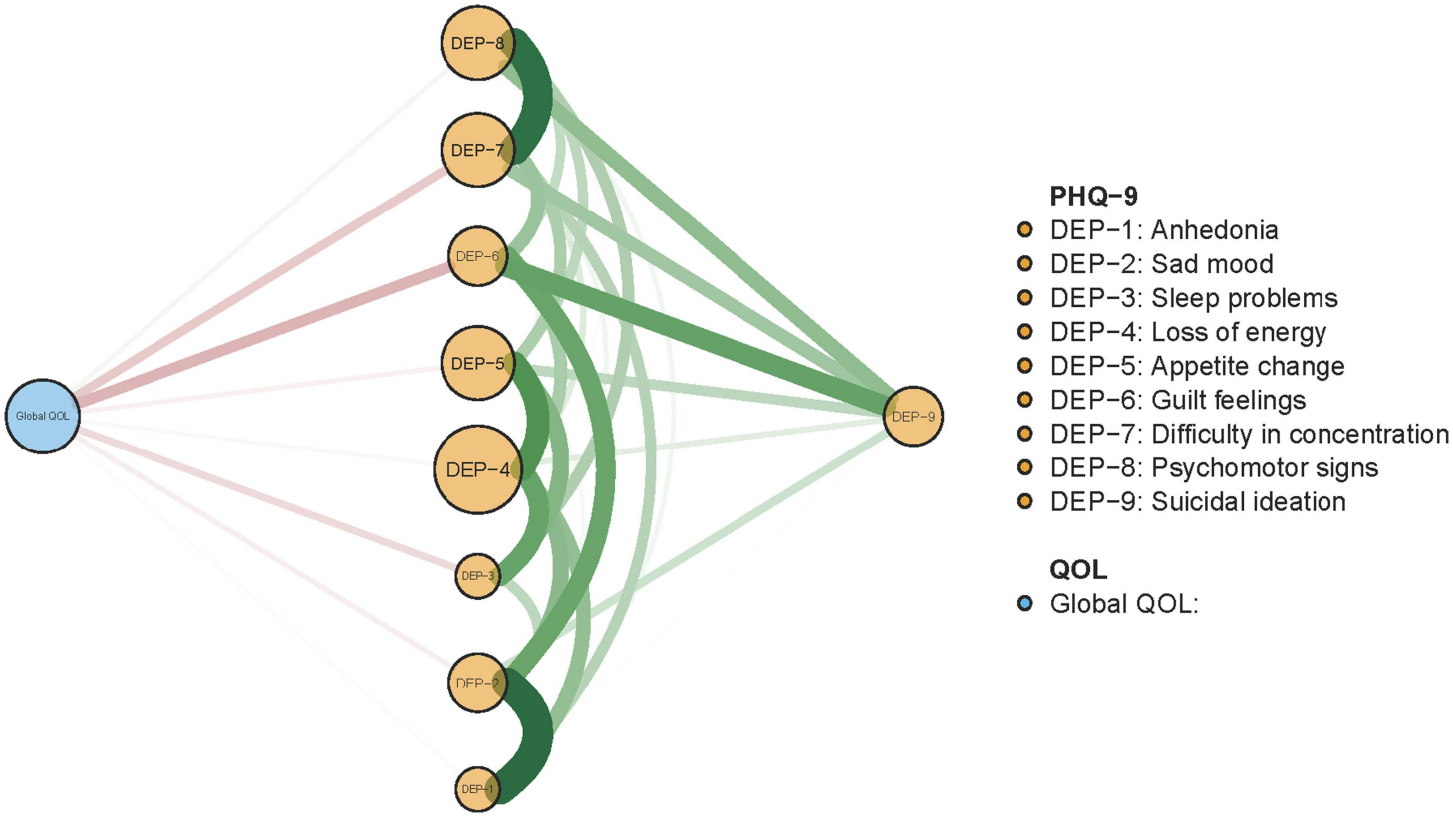
Flow network of QOL and depressive symptoms among guardians of hospitalized psychiatric patients (*N* = 1,101).

The re-estimated network structure of depressive symptoms after adjusting for anxiety symptoms and fatigue is shown in Figure 3. Nodes with three highest strengths in the adjusted network (Figure 3) were identical to those in the unadjusted network (Figure 1), suggesting that neither anxiety symptoms nor fatigue had a significant influence on the initial network model. Exact centrality strengths in the adjusted network model are shown in Supplementary Table S3. The flow network of depressive symptoms and QOL indicated PHQ-9 items 6 (DEP-6, guilt feelings), 7 (DEP-7, concentration difficulties) and 3 (DEP-3, sleep problems) were strongly connected with global QOL within the overall guardian sample (Figure 4). The weighted adjacency matrix of the network for global QOL and depressive symptoms was shown in Supplementary Table S4.

Supplementary subgroup network analyses showed that the network features in depressed guardians were similar to those found for the whole sample (Supplementary Figures S1, S2).

## 4. Discussion

To our knowledge, this is the first study to explore the prevalence of depression and its association with QOL among guardians of hospitalized psychiatric patients during the COVID-19 pandemic. The prevalence of depression among guardians was 32.4%. Since no prevalence data from previous studies of guardians of hospitalized psychiatric patients could be identified, it is not entirely clear whether the rate in this sample was elevated relative to related comparison groups. However, previous COVID-19 pandemic era studies (61, 62) on guardians to assisted living residents and guardians to persons with neurocognitive disorders reported rates of depression (38.8% and 36.3% respectively) similar to those of the present study. Given that approximately one third of guardians experienced depression across these three studies, depression among caregivers of vulnerable patient groups appears to be a noteworthy yet overlooked mental health problem during the COVID-19 pandemic.

The relatively high prevalence of depression among guardians in this study could be attributed to several reasons. First, the closure of clinical psychiatric services during the early COVID-19 pandemic phase could have contributed acute patient crises (8, 63), including difficulties in visiting psychiatrists, reduced access to psychotropic medications, and/or barriers in maintaining medication compliance, all of which could exacerbate distress in patients as well as concerned family members including guardians. Second, news reports of increased nosocomial infections of COVID-19 within psychiatric hospitals could have aggravated guardians’ pandemic-related worries (10). Third, cancellations of routine family visits to hospitals during the COVID-19 pandemic increased uncertainty about care for both patients and guardians. Finally, the PHQ-9 cutoff we adopted to identify depressed status may have contributed to this rate and is not necessarily identical to prevalence estimates that might be garnered from structured diagnostic interviews.

With respect to unique predictors of depression among guardians in our sample, higher GAD-7 total scores were positively correlated with depression scores. This finding aligns with previous studies indicating anxiety and depression are frequently comorbid with each other (64, 65). To elaborate, a worldwide survey reported that almost 46% of patients with a lifetime prevalence of major depressive disorder (MDD) also have a lifetime history of anxiety disorder (66). Data from the Sequenced Treatment Alternatives to Relieve Depression (STAR*D) study found 53% of patients with MDD had significant concurrent anxiety symptoms (67). Depression and anxiety are also intertwined with one another over time (68); the presence of one condition may predispose the vulnerable to the other condition (69). Supporting biological foundations of comorbidity, genetic epidemiological studies suggest that depression and anxiety have a shared genetic etiology (70–73).

High levels of fatigue also emerged as a unique correlate of elevated depression scores in our sample. Paralleling comorbidity evidence for anxiety, fatigue is often viewed as comorbid with depression and is highly prevalent in a cluster of depressive symptoms (74–76), particularly within East-Asian samples who may somatize depressive symptoms (77). Neural pathway studies have also found chronic fatigue and depression have shared neurobiological mechanisms (78, 79). In contrast to comorbidity interpretations, associations between depression and fatigue may be attributed to construct overlaps. Specifically, the diagnosis of depression and PHQ-9 include “loss of energy” as a criterion (36, 37) that overlaps with fatigue.

Network analysis indicated “*loss of energy”* (DEP-4) had the highest centrality strength in the structure of depressive symptoms in our guardian sample. This finding aligns with Hinz et al. (80) who reported “loss of energy” had the highest factor loading of any PHQ-9 item. In tandem, these results underscore the importance of loss of energy vis a vis other symptoms of depression. In community-based settings, “*loss of energy”* is frequently endorsed when people encounter depressing life events (81, 82). Conversely, in psychiatric samples, the most central symptom is often “*sad mood*” (83, 84). This discrepancy highlights potential differences in the expression of depression between psychiatric and non-psychiatric samples such as guardians in this study. “Loss of energy” may be more central to experiences of depression among guardians of hospitalized psychiatric patients, in part, due to adopting a less physically active lifestyle during lockdowns (85, 86) and/or increased stress associated with potentially heavier caregiving burdens related to fulfilling the guardian role during a pandemic (87).

“C*oncentration difficulties”* (DEP-7) had the second highest strength centrality in the network of depressive symptoms in our guardian sample. “*Sad mood*” and “*anhedonia*” are conventionally accepted as two core symptoms of MDD, in contrast to our finding that “*concentration difficulties*” emerged as the second most influential depressive symptom in guardians of hospitalized psychiatric patients. This could be explained, in part, by the fact that the PHQ-9 is a screening measure on depressive symptoms based on continuous severity ratings, rather than an MDD diagnosis. Nonetheless, more influential symptoms in the network model of depressive symptoms based on the PHQ-9 assessment align with symptoms of MDD based on DSM criteria as well as research based on samples with similar characteristics. Specifically, our centrality influence findings are consistent with a previous study in which individuals with an external locus of attribution were more vulnerable to concentration problems than those with an internal locus of attribution (88). A comparatively stronger external orientation may help to explain the centrality of “*concentration difficulties*” (DEP-7) in the network model of depressive symptoms among guardians since extra guardianship and caregiving responsibilities of this group may have increased the likelihood of emphasizing external influences as causes of stress experiences. Moreover, concentration problems may be more prominent when levels of depression severity are low (89); presumably, a majority of guardians in our study sample did not experience severe depression in light of the need for considerable competence in undertaking their role. Our data suggest that “concentration difficulties” could be an important yet easily overlooked indicator in populations that experience stress and undertake guardianship or caregiving responsibilities.

“S*ad mood*” (DEP-2) had the third highest strength centrality in the network model of depression among guardians in this study. This finding converges with evidence from Cheung et al.’s (90) network structure study of depressive symptoms in a community sample from Hong Kong during the COVID-19 pandemic, Hartung et al.’s (91) network analysis of PHQ-9 items in a sample from the general population in Germany, and Fried et al.’s (92) study of depressive symptomatology in outpatients with MDD. Despite differences in sample characteristics, these studies highlight sad mood as a central symptom of depression even when samples are relatively high functioning.

Finally, after adjusting for significant correlates of depression including anxiety and fatigue, depressed guardians had significantly lower QOL levels than their non-depressed peers did. The negative depression-QOL association appears to be robust given that it has also been observed in other populations including community-dwellers, older persons, and patients with cancer (93–96). From a symptom-level perspective, “*guilt feelings*” (DEP-6), “*concentration difficulties*” (DEP-7) and “*sleep problems*” (DEP-3) had the strongest associations with global QOL in our guardian sample. As such, these symptoms could be useful targets for interventions designed to alleviate depression and improve QOL in this population.

Strengths of this study included its relatively large sample size, multi-center study design, and adoption of both a broad epidemiological perspective and a symptom-level perspective to evaluate depressive symptoms within an understudied population involved in the care of patients with psychiatric disorders. However, the study also had several methodological limitations. First, because a cross-sectional design was used, the time course of depression and changes in the expression of individual depressive symptoms over different phases of the pandemic could not be elucidated. On a related note, pre-versus post-pandemic rates of depression and network models could not be assessed due to the cross-sectional design and initiation of this study only after the COVID-19 pandemic had begun. Third, the network structure of depression was limited to PHQ-9 items so it is possible that the network structure might differ based on a different depression questionnaire or interview-based assessment. Fourth, although WeChat is widely used in China and all guardians were presumed to be WeChat users, recruitment based on consecutive (i.e., non-probability sampling) rather than random sampling, is more prone to selection biases. Finally, it is not clear how well our findings extend to guardian samples in other countries that have experienced high levels of morbidity and mortality from COVID-19 and have adopted different policies for managing the pandemic.

In conclusion, this study found approximately 1/3 of guardians of hospitalized psychiatric patients in China reported depression during the COVID-19 pandemic. Anxiety and fatigue emerged as unique correlates of depression in the sample. “*Loss of energy”* (DEP-4), “*concentration difficulties”* (DEP-7), and “*sad mood”* (DEP-2) were the most influential symptoms in the associated network model. These symptoms could be valuable targets in treatments for depression while strategies to reduce sleep problems and guilt may aid in improving QOL of guardians.

## Data availability statement

The datasets presented in this article are not readily available because the Research Ethics Committee of Beijing Anding Hospital that approved the study prohibits the authors from making publicly available the research dataset of clinical studies. Requests to access the datasets should be directed to xyutly@gmail.com.

## Ethics statement

The studies involving human participants were reviewed and approved by Research Ethics Committee of Beijing Anding Hospital, Capital Medical University. The patients/participants provided their electronic written informed consent to participate in this study.

## Author contributions

Y-JZ, LZ, YF, SS, and Y-TX: study design. Y-JZ, LZ, YF, SS, ML, Y-YW, J-XL, ZS, TC, and GU: data collection, analysis, and interpretation. Y-JZ and Y-TX: drafting of the manuscript. TJ: critical revision of the manuscript. All authors contributed to the article and approved the submitted version.

## Funding

This study was supported by Beijing Hospitals Authority Youth Programme (code: 1-1-2-2-xm202301-01), Beijing Municipal Science & Technology Commission (No. Z181100001518005), the National Science and Technology Major Project for investigational new drug (2018ZX09201-014), the Beijing Hospitals Authority Clinical Medicine Development of special funding support (XMLX202128), and the University of Macau (MYRG2019-00066-FHS and MYRG2022-00187-FHS).

## Conflict of interest

The authors declare that the research was conducted in the absence of any commercial or financial relationships that could be construed as a potential conflict of interest.

## Publisher’s note

All claims expressed in this article are solely those of the authors and do not necessarily represent those of their affiliated organizations, or those of the publisher, the editors and the reviewers. Any product that may be evaluated in this article, or claim that may be made by its manufacturer, is not guaranteed or endorsed by the publisher.

## References

[1] World Health Organization. Naming the coronavirus disease (COVID-19) and the virus that causes it. Available at: https://wwwwhoint/emergencies/diseases/novel-coronavirus-2019/technical-guidance/naming-the-coronavirus-disease-(covid-2019)-and-the-virus-that-causes-it (Accessed February 11, 2020) (2020).

[2] World Health Organization. Novel coronavirus – China. Availabel at: (https://wwwwhoint/csr/don/12-january-2020-novel-coronavirus-china/en/) (2020).

[3] Zhao YJ Zhang SF Li W Zhang L Guo T Cheung T. Associations between depressive symptoms and quality of life among residents of Wuhan, China during the later stage of the COVID-19 pandemic: a network analysis. J Affect Disord. (2022) 318:456–64. doi: 10.1016/j.jad.2022.08.104, PMID: 36058363 PMC9436879

[4] Kılınçel Ş Kılınçel O Muratdağı G Aydın A Usta MB. Factors affecting the anxiety levels of adolescents in home-quarantine during Covid-19 pandemic in Turkey. Asia Pac Psychiatry. (2021) 13:e12406. doi: 10.1111/appy.12406, PMID: 32783389 PMC7435562

[5] Li T Sun S Liu B Wang J Zhang Y Gong C. Prevalence and risk factors for anxiety and depression in patients with Covid-19 in Wuhan. China Psychosom Med. (2021) 83:368–72. doi: 10.1097/psy.0000000000000934 33951724

[6] Seminog OO Goldacre MJ. Risk of pneumonia and pneumococcal disease in people with severe mental illness: English record linkage studies. Thorax. (2013) 68:171–6. doi: 10.1136/thoraxjnl-2012-202480, PMID: 23242947

[7] Xiang YT Zhao YJ Liu ZH Li XH Zhao N Cheung T. The COVID-19 outbreak and psychiatric hospitals in China: managing challenges through mental health service reform. Int J Biol Sci. (2020) 16:1741–4. Epub 2020/04/01. doi: 10.7150/ijbs.45072, PMID: 32226293 PMC7098035

[8] Li L. Challenges and priorities in responding to Covid-19 in inpatient psychiatry. Psychiatr Serv. (2020) 71:624–6. doi: 10.1176/appi.ps.202000166, PMID: 32321388

[9] Shinn AK Viron M. Perspectives on the Covid-19 pandemic and individuals with serious mental illness. J Clin Psychiatry. (2020) 81. doi: 10.4088/JCP.20com13412 32369691

[10] China News Weekly. Hospital-acquired infection in Wuhan mental health center: around 80 medical staff and patients were diagnosed with 2019-Ncov pneumonia (in Chinese). Available at: (https://newssinacomcn/c/2020-02-08/doc-iimxxste9892538shtml) (2020).

[11] Wang Q Xu R Volkow ND. Increased risk of COVID-19 infection and mortality in people with mental disorders: analysis from electronic health records in the United States. World Psychiatry. (2021) 20:124–30. doi: 10.1002/wps.20806, PMID: 33026219 PMC7675495

[12] Iftimie S López-Azcona AF Lozano-Olmo MJ Hernández-Aguilera A Sarrà-Moretó S Joven J. Characteristics of hospitalized patients with Sars-Cov-2 infection during successive waves of the Covid-19 pandemic in a reference Hospital in Spain. Sci Rep. (2022) 12:17384. doi: 10.1038/s41598-022-22145-9, PMID: 36253391 PMC9574827

[13] Diez-Quevedo C Iglesias-González M Giralt-López M Rangil T Sanagustin D Moreira M. Mental disorders, psychopharmacological treatments, and mortality in 2150 COVID-19 Spanish inpatients. Acta Psychiatr Scand. (2021) 143:526–34. doi: 10.1111/acps.13304, PMID: 33792912 PMC8250711

[14] Bojdani E Rajagopalan A Chen A Gearin P Olcott W Shankar V. COVID-19 pandemic: impact on psychiatric care in the United States. Psychiatry Res. (2020) 289:113069. doi: 10.1016/j.psychres.2020.113069, PMID: 32413707 PMC7200362

[15] Li S Zhang Y. Mental healthcare for psychiatric inpatients during the COVID-19 epidemic. Gen Psychiatr. (2020) 33:e100216. doi: 10.1136/gpsych-2020-100216, PMID: 32363326 PMC7174023

[16] Xie Q Fan F Fan XP Wang XJ Chen MJ Zhong BL. COVID-19 patients managed in psychiatric inpatient settings due to first-episode mental disorders in Wuhan, China: clinical characteristics, treatments, outcomes, and our experiences. Transl Psychiatry. (2020) 10:337. doi: 10.1038/s41398-020-01022-x, PMID: 33009366 PMC7531059

[17] Cui LB Wang XH Wang HN. Challenges of facing coronavirus disease 2019: psychiatric services for patients with mental disorders. Psychiatry Clin Neurosci. (2020) 74:371–2. doi: 10.1111/pcn.13003, PMID: 32237013

[18] Dorman-Ilan S Hertz-Palmor N Brand-Gothelf A Hasson-Ohayon I Matalon N Gross R. Anxiety and depression symptoms in COVID-19 isolated patients and in their relatives. Front Psych. (2020) 11:581598. doi: 10.3389/fpsyt.2020.581598, PMID: 33192727 PMC7591814

[19] Czeisler M Rohan EA Melillo S Matjasko JL DePadilla L Patel CG. Mental health among parents of children aged <18 years and unpaid caregivers of adults during the COVID-19 pandemic – United States, December 2020 and February-⁠march 2021. MMWR Morb Mortal Wkly Rep. (2021) 70:879–87. doi: 10.15585/mmwr.mm7024a3 34138835 PMC8220951

[20] Alessi J de Oliveira GB Feiden G Schaan BD Telo GH. Caring for caregivers: the impact of the COVID-19 pandemic on those responsible for children and adolescents with type 1 diabetes. Sci Rep. (2021) 11:6812. doi: 10.1038/s41598-021-85874-3, PMID: 33762633 PMC7991637

[21] Reece L Sams DP. The impact of Covid-19 on adolescent psychiatric inpatient admissions. Clin Child Psychol Psychiatry. (2022) 27:112–21. doi: 10.1177/13591045211030666, PMID: 34229484 PMC8689095

[22] Hu Y Chen Y Zheng Y You C Tan J Hu L. Factors related to mental health of inpatients with COVID-19 in Wuhan, China. Brain Behav Immun. (2020) 89:587–93. doi: 10.1016/j.bbi.2020.07.016, PMID: 32681866 PMC7362867

[23] Yalçin M Sönmez Güngör E Ergelen M Beşikçi Keleş D Yerebakan Tüzer M Öcek Baş T. Characteristics and outcomes of psychiatric inpatients with severe mental illness and COVID-19: experience from a COVID-19-specific acute psychiatric Ward in Istanbul. J Nerv Ment Dis. (2021) 209:884–91. doi: 10.1097/nmd.0000000000001450, PMID: 34710895 PMC8614197

[24] Yao H Chen JH Xu YF. Patients with mental health disorders in the Covid-19 epidemic. Lancet Psychiatry. (2020) 7:e21. doi: 10.1016/s2215-0366(20)30090-0, PMID: 32199510 PMC7269717

[25] Everett B. An introduction to latent variable models Springer Science & Business Media (2013).

[26] Schmittmann VD Cramer AO Waldorp LJ Epskamp S Kievit RA Borsboom D. Deconstructing the construct: a network perspective on psychological phenomena. New Ideas Psychol. (2013) 31:43–53. doi: 10.1016/j.newideapsych.2011.02.007

[27] Borsboom D. Psychometric perspectives on diagnostic systems. J Clin Psychol. (2008) 64:1089–108. doi: 10.1002/jclp.20503 18683856

[28] Santos H Jr Fried EI Asafu-Adjei J Ruiz RJ. Network structure of perinatal depressive symptoms in Latinas: relationship to stress and reproductive biomarkers. Res Nurs Health. (2017) 40:218–28. doi: 10.1002/nur.21784, PMID: 28220506 PMC5503306

[29] Marchetti I. Hopelessness: a network analysis. Cogn Ther Res. (2018) 43:611–9. doi: 10.1007/s10608-018-9981-y

[30] Mullarkey MC Marchetti I Beevers CG. Using network analysis to identify central symptoms of adolescent depression. J Clin Child Adolesc Psychol. (2019) 48:656–68. doi: 10.1080/15374416.2018.1437735, PMID: 29533089 PMC6535368

[31] Borsboom D. A network theory of mental disorders. World Psychiatry. (2017) 16:5–13. doi: 10.1002/wps.20375, PMID: 28127906 PMC5269502

[32] Borsboom D Cramer AO. Network analysis: An integrative approach to the structure of psychopathology. Annu Rev Clin Psychol. (2013) 9:91–121. doi: 10.1146/annurev-clinpsy-050212-185608 23537483

[33] Fried EI Nesse RM. Depression sum-scores don't add up: why analyzing specific depression symptoms is essential. BMC Med. (2015) 13:72. doi: 10.1186/s12916-015-0325-4, PMID: 25879936 PMC4386095

[34] Liu J Zhu Q Fan W Makamure J Zheng C Wang J. Online mental health survey in a medical College in China during the COVID-19 outbreak. Front Psych. (2020) 11:459. doi: 10.3389/fpsyt.2020.00459, PMID: 32574242 PMC7237734

[35] Liu Y Zhang Z Zhao H. The influence of the Covid-19 event on deviant workplace behavior taking Tianjin, Beijing and Hebei as an example. Int J Environ Res Public Health. (2020) 18. doi: 10.3390/ijerph18010059 PMC7794894 33374789

[36] Spitzer RL Kroenke K Williams JB. Validation and utility of a self-report version of prime-md: the PHQ primary care study. Primary care evaluation of mental disorders. Patient health questionnaire. JAMA. (1999) 282:1737–44. doi: 10.1001/jama.282.18.1737, PMID: 10568646

[37] Kroenke K Spitzer RL Williams JB. The PHQ-9: validity of a brief depression severity measure. J Gen Intern Med. (2001) 16:606–13. doi: 10.1046/j.1525-1497.2001.016009606.x, PMID: 11556941 PMC1495268

[38] Chen MM Sheng L Qu S. Diagnostic test of screening depressive disorder in general hospital with the patient health questionnaire (in Chinese). J Chinese Mental Health. (2015) 29:241–5. doi: 10.3969/j.issn.1000-6729.2015.04.001

[39] Wang W Bian Q Zhao Y Li X Wang W Du J. Reliability and validity of the Chinese version of the patient health questionnaire (PHQ-9) in the general population. Gen Hosp Psychiatry. (2014) 36:539–44. doi: 10.1016/j.genhosppsych.2014.05.021, PMID: 25023953

[40] Xu Y Wu HS Xu YF. The application of Patient Health Questionnaire 9 in community elderly population: reliability and validity (in Chinese). Shanghai Arch Psychiatry. (2007) 19:257.

[41] Spitzer RL Kroenke K Williams JB Löwe B. A brief measure for assessing generalized anxiety disorder: the GAD-7. Arch Intern Med. (2006) 166:1092–7. doi: 10.1001/archinte.166.10.1092 16717171

[42] Zhang C Wang T Zeng P Zhao M Zhang G Zhai S. Reliability, validity, and measurement invariance of the general anxiety disorder scale among Chinese medical university students. Front Psych. (2021) 12:648755. doi: 10.3389/fpsyt.2021.648755 PMC8170102 34093269

[43] Zeng Q-Z He Y-L Liu H Miao J-M Chen J-X Xu H-N. Reliability and validity of Chinese version of the Generalized Anxiety Disorder 7-item (GAD-7) scale in screening anxiety disorders in outpatients from traditional Chinese internal department (in Chinese). Chin Ment Health J. (2013) 27:163–8.

[44] Gladman D Nash P Goto H Birt JA Lin CY Orbai AM. Fatigue numeric rating scale validity, discrimination and responder definition in patients with psoriatic arthritis. RMD Open. (2020) 6:e000928. doi: 10.1136/rmdopen-2019-000928, PMID: 31958274 PMC7046948

[45] Fang J-Q Hao Y-T Li C-X. Reliability and validity for Chinese version of who quality of life scale (in Chinese). Chinese. J Ment Health. (1999) 13

[46] The WHOQOL GROUP. Development of the World Health Organization WHOQOL-BREF quality of life assessment. The WHOQOL Group. Psychol Med. (1998) 28:551–8. doi: 10.1017/s0033291798006667 9626712

[47] Skevington SM Tucker C. Designing response scales for cross-cultural use in health care: data from the development of the UK WHOQOL. Br J Med Psychol. (1999) 72:51–61. doi: 10.1348/000711299159817, PMID: 10194572

[48] Xia P Li N Hau KT Liu C Lu Y. Quality of life of Chinese Urban Community residents: a psychometric study of the mainland Chinese version of the WHOQOL-BREF. BMC Med Res Methodol. (2012) 12:37. doi: 10.1186/1471-2288-12-37, PMID: 22452994 PMC3364902

[49] R Core Team. R: A language and environment for statistical computing. Vienna, Austria: R Foundation for Statistical Computing (2020).

[50] Zhao YJ Xing X Tian T Wang Q Liang S Wang Z. Post Covid-19 mental health symptoms and quality of life among COVID-19 frontline clinicians: a comparative study using propensity score matching approach. Transl Psychiatry. (2022) 12:376. doi: 10.1038/s41398-022-02089-4, PMID: 36085292 PMC9461449

[51] Bai W Zhao YJ Cai H Sha S Zhang Q Lei SM. Network analysis of depression, anxiety, insomnia and quality of life among Macau residents during the COVID-19 pandemic. J Affect Disord. (2022) 311:181–8. doi: 10.1016/j.jad.2022.05.061, PMID: 35594975 PMC9112609

[52] Di Blasi M Gullo S Mancinelli E Freda MF Esposito G Gelo OCG. Psychological distress associated with the COVID-19 lockdown: a two-wave network analysis. J Affect Disord. (2021) 284:18–26. doi: 10.1016/j.jad.2021.02.016, PMID: 33582428 PMC8771473

[53] Chernick MR. Bootstrap methods: A guide for practitioners and researchers. 2nd ed. New Jersey: John Wiley & Sons (2008). 619 p.

[54] Costenbader E Valente TW. The stability of centrality measures when networks are sampled. Soc Networks. (2003) 25:283–307. doi: 10.1016/S0378-8733(03)00012-1

[55] Epskamp S Borsboom D Fried EI. Estimating psychological networks and their accuracy: a tutorial paper. Behav Res Methods. (2018) 50:195–212. doi: 10.3758/s13428-017-0862-1, PMID: 28342071 PMC5809547

[56] Jones P. Networktools: tools for identifying important nodes in networks. R package version 1.2.3. Available at: https://CRANR-projectorg/package=networktools (2020).

[57] Epskamp S Cramer AO Waldorp LJ Schmittmann VD Borsboom D. qgraph: network visualizations of relationships in psychometric data. J Stat Softw. (2012) 48:1–18.

[58] van Borkulo C Boschloo L Kossakowski J Tio P Schoevers R Borsboom D. Comparing network structures on three aspects: a permutation test. J Stat Softw. (2017). doi: 10.13140/RG.2.2.29455.38569 35404628

[59] van Borkulo CD van Bork R Boschloo L Kossakowski JJ Tio P Schoevers RA. Comparing network structures on three aspects: a permutation test. Psychol Methods. (2022). doi: 10.1037/met0000476 35404628

[60] Haslbeck J Waldorp LJ. mgm: estimating time-varying mixed graphical models in high-dimensional data. J Stat Softw. (2020) 93:1–46. doi: 10.18637/jss.v093.i08

[61] Lane NE Hoben M Amuah JE Hogan DB Baumbusch J Gruneir A. Prevalence and correlates of anxiety and depression in caregivers to assisted living residents during Covid-19: a cross-sectional study. BMC Geriatr. (2022) 22:662. doi: 10.1186/s12877-022-03294-y, PMID: 35962356 PMC9372518

[62] Li Q Zhang H Zhang M Li T Ma W An C. Prevalence and risk factors of anxiety, depression, and sleep problems among caregivers of people living with neurocognitive disorders during the COVID-19 pandemic. Front Psych. (2020) 11:590343. doi: 10.3389/fpsyt.2020.590343, PMID: 33488423 PMC7820074

[63] Souza ALR Guimarães RA de Araújo VD de Assis RM de Almeida Cavalcante Oliveira LM Souza MR. Factors associated with the burden of family caregivers of patients with mental disorders: a cross-sectional study. BMC Psychiatry. (2017) 17:353. doi: 10.1186/s12888-017-1501-1 29070012 PMC5655908

[64] Haug TT Mykletun A Dahl AA. The association between anxiety, depression, and somatic symptoms in a large population: the hunt-ii study. Psychosom Med. (2004) 66:845–51. doi: 10.1097/01.psy.0000145823.85658.0c, PMID: 15564348

[65] Ligthart L Gerrits MM Boomsma DI Penninx BW. Anxiety and depression are associated with migraine and pain in general: an investigation of the interrelationships. J Pain. (2013) 14:363–70. doi: 10.1016/j.jpain.2012.12.006, PMID: 23395476

[66] Kessler RC Sampson NA Berglund P Gruber MJ Al-Hamzawi A Andrade L. Anxious and non-anxious major depressive disorder in the World Health Organization world mental health surveys. Epidemiol Psychiatr Sci. (2015) 24:210–26. doi: 10.1017/s2045796015000189, PMID: 25720357 PMC5129607

[67] Fava M Alpert JE Carmin CN Wisniewski SR Trivedi MH Biggs MM. Clinical correlates and symptom patterns of anxious depression among patients with major depressive disorder in Star*D. Psychol Med. (2004) 34:1299–308. doi: 10.1017/s0033291704002612, PMID: 15697056

[68] Jansson-Fröjmark M Lindblom K. A bidirectional relationship between anxiety and depression, and insomnia? A prospective study in the general population. J Psychosom Res. (2008) 64:443–9. doi: 10.1016/j.jpsychores.2007.10.016, PMID: 18374745

[69] Frances A Manning D Marin D Kocsis J McKinney K Hall W. Relationship of anxiety and depression. Psychopharmacology. (1992) 106:S82–6. doi: 10.1007/BF02246243 1546149

[70] Hettema JM. What is the genetic relationship between anxiety and depression? Am J Med Genet C Semin Med Genet. (2008) 148c:140–6. doi: 10.1002/ajmg.c.30171 18412101

[71] Sullivan PF Neale MC Kendler KS. Genetic epidemiology of major depression: review and meta-analysis. Am J Psychiatry. (2000) 157:1552–62. doi: 10.1176/appi.ajp.157.10.1552 11007705

[72] Hettema JM Neale MC Kendler KS. A review and meta-analysis of the genetic epidemiology of anxiety disorders. Am J Psychiatry. (2001) 158:1568–78. doi: 10.1176/appi.ajp.158.10.1568 11578982

[73] Middeldorp CM Cath DC Van Dyck R Boomsma DI. The co-morbidity of anxiety and depression in the perspective of genetic epidemiology. A review of twin and family studies. Psychol Med. (2005) 35:611–24. doi: 10.1017/s003329170400412x, PMID: 15918338

[74] Corfield EC Martin NG Nyholt DR. Co-occurrence and symptomatology of fatigue and depression. Compr Psychiatry. (2016) 71:1–10. doi: 10.1016/j.comppsych.2016.08.004, PMID: 27567301

[75] Pae C-U Lim H-K Han C Patkar AA Steffens DC Masand PS. Fatigue as a Core symptom in major depressive disorder: overview and the role of bupropion. Expert Rev Neurother. (2007) 7:1251–63. doi: 10.1586/14737175.7.10.1251, PMID: 17939764

[76] Reyes-Gibby CC Aday LA Anderson KO Mendoza TR Cleeland CS. Pain, depression, and fatigue in community-dwelling adults with and without a history of cancer. J Pain Symptom Manage. (2006) 32:118–28. doi: 10.1016/j.jpainsymman.2006.01.008, PMID: 16877179 PMC1950719

[77] Stahl SM. Does depression hurt? J Clin Psychiatry. (2002) 63:273–4. doi: 10.4088/JCP.v63n0401 12000200

[78] Berman RM Narasimhan M Miller HL Anand A Cappiello A Oren DA. Transient depressive relapse induced by catecholamine depletion: potential phenotypic vulnerability marker? Arch Gen Psychiatry. (1999) 56:395–403. doi: 10.1001/archpsyc.56.5.395, PMID: 10232292

[79] Chaves-Filho AJM Macedo DS de Lucena DF Maes M. Shared microglial mechanisms underpinning depression and chronic fatigue syndrome and their comorbidities. Behav Brain Res. (2019) 372:111975. doi: 10.1016/j.bbr.2019.111975, PMID: 31136774

[80] Hinz A Mehnert A Kocalevent R-D Brähler E Forkmann T Singer S. Assessment of depression severity with the PHQ-9 in cancer patients and in the general population. BMC Psychiatry. (2016) 16:1–8. 26831145 10.1186/s12888-016-0728-6 PMC4736493

[81] Fava GA Tossani E. Prodromal stage of major depression. Early Interv Psychiatry. (2007) 1:9–18. doi: 10.1111/j.1751-7893.2007.00005.x 21352104

[82] Robinson RL Stephenson JJ Dennehy EB Grabner M Faries D Palli SR. The importance of unresolved fatigue in depression: costs and comorbidities. Psychosomatics. (2015) 56:274–85. doi: 10.1016/j.psym.2014.08.003, PMID: 25596022

[83] Beard C Millner AJ Forgeard MJ Fried EI Hsu KJ Treadway MT. Network analysis of depression and anxiety symptom relationships in a psychiatric sample. Psychol Med. (2016) 46:3359–69. doi: 10.1017/S0033291716002300, PMID: 27623748 PMC5430082

[84] van Rooijen G Isvoranu AM Kruijt OH van Borkulo CD Meijer CJ Wigman JTW. A state-independent network of depressive, negative and positive symptoms in male patients with schizophrenia spectrum disorders. Schizophr Res. (2018) 193:232–9. doi: 10.1016/j.schres.2017.07.035, PMID: 28844638

[85] McIlvenny S DeGlume A Elewa M Fernandez O Dormer P. Factors associated with fatigue in a family medicine clinic in the United Arab Emirates. Fam Pract. (2000) 17:408–13. doi: 10.1093/fampra/17.5.408, PMID: 11021901

[86] Stewart D Abbey S Meana M Boydell KM. What makes women tired? A Community Sample J Womens Health. (1998) 7:69–76. doi: 10.1089/jwh.1998.7.69, PMID: 9511134

[87] Sharpe M Wilks D. Fatigue. BMJ. (2002) 325:480–3. doi: 10.1136/bmj.325.7362.480, PMID: 12202331 PMC1124000

[88] Bekhuis E Olde Hartman TC Boschloo L Lucassen PL. A new approach to psychopathology: the example of depression. Br J Gen Pract. (2019) 69:146–7. doi: 10.3399/bjgp19X701717, PMID: 30819756 PMC6400597

[89] Cole DA Cai L Martin NC Findling RL Youngstrom EA Garber J. Structure and measurement of depression in youths: applying item response theory to clinical data. Psychol Assess. (2011) 23:819–33. doi: 10.1037/a0023518, PMID: 21534696 PMC3743727

[90] Cheung T Jin Y Lam S Su Z Hall BJ Xiang YT. Network analysis of depressive symptoms in Hong Kong residents during the COVID-19 pandemic. Transl Psychiatry. (2021) 11:460. doi: 10.1038/s41398-021-01543-z, PMID: 34489416 PMC8419676

[91] Hartung TJ Fried EI Mehnert A Hinz A Vehling S. Frequency and network analysis of depressive symptoms in patients with cancer compared to the general population. J Affect Disord. (2019) 256:295–301. doi: 10.1016/j.jad.2019.06.009, PMID: 31200167

[92] Fried EI Epskamp S Nesse RM Tuerlinckx F Borsboom D. What are 'Good' depression symptoms? Comparing the centrality of DSM and non-DSM symptoms of depression in a network analysis. J Affect Disord. (2016) 189:314–20. doi: 10.1016/j.jad.2015.09.005, PMID: 26458184

[93] Lépine JP Briley M. The increasing burden of depression. Neuropsychiatr Dis Treat. (2011) 7:3–7. doi: 10.2147/ndt.S19617, PMID: 21750622 PMC3131101

[94] Sivertsen H Bjørkløf GH Engedal K Selbæk G Helvik AS. Depression and quality of life in older persons: a review. Dement Geriatr Cogn Disord. (2015) 40:311–39. doi: 10.1159/000437299, PMID: 26360014

[95] Lou Z Li Y Yang Y Wang L Yang J. Affects of anxiety and depression on health-related quality of life among patients with benign breast lumps diagnosed via ultrasonography in China. Int J Environ Res Public Health. (2015) 12:10587–601. doi: 10.3390/ijerph120910587, PMID: 26343700 PMC4586630

[96] Tan EJ Rossell SL. Comparing how co-morbid depression affects individual domains of functioning and life satisfaction in schizophrenia. Compr Psychiatry. (2016) 66:53–8. doi: 10.1016/j.comppsych.2015.12.007, PMID: 26995236

