# Supplementary material for: Prevalence of depression and its association with quality of life among guardians of hospitalized psychiatric patients during the COVID-19 pandemic: a network perspective
Source: Front Psychiatry. 2023 May 12;14:1139742. doi: 10.3389/fpsyt.2023.1139742 (PMC10213336; doi:10.3389/fpsyt.2023.1139742)
Supplement: Supplementary file 1 [file Data_Sheet_1.docx]

**Supplementary material**

Supplementary Table 1. Independent correlates of depression among first degree relatives of hospitalized psychiatric patients during the COVID-19 pandemic (N=792)

Supplementary Table 2. Strengths of the depressive symptoms among guardians of hospitalized psychiatric patients

Supplementary Table 3. Strengths of the depressive symptoms among guardians of hospitalized psychiatric patients after adjusting for anxiety symptoms and fatigue

Supplementary Table 4. Weighted adjacency matrix of the network for global QOL and depressive symptoms among guardians of hospitalized psychiatric patients

Supplementary Figure 1. Comparison of the network structures for depressive symptoms between the whole sample (N=1,101) and the depressed subgroup (N=357)

Supplementary Figure 2. Network invariance test and global strength test of the network structures for depressive symptoms between the whole sample (N=1,101) and the depressed subgroup (N=357)

Supplementary Figure 3. Network stability of depressive symptoms among depressed guardians of hospitalized psychiatric patients (N=357)

Supplementary Table 1. Independent correlates of depression among first degree relatives of hospitalized psychiatric patients during the COVID-19 pandemic (N=792)

| Variables | Multiple logistic regression analysis | | |
| --- | --- | --- | --- |
|  | *p* | *OR* | 95% *CI* |
| Age (years) | 0.92 | 1.0 | 0.98-1.02 |
| Female | 0.53 | 0.9 | 0.5-1.4 |
| Presence of major physical diseases | 0.87 | 1.1 | 0.4-3.2 |
| Perceived financial status (poor vs. fair/good) | 0.23 | 1.4 | 0.8-2.4 |
| Frequency of social media use (often vs. no or minimal/sometimes) | 0.31 | 0.8 | 0.5-1.3 |
| Difficulty in visiting mental health service during the pandemic | 0.32 | 1.3 | 0.8-2.2 |
| GAD-7 total | **<0.001** | 1.9 | 1.7-2.0 |
| Fatigue | **<0.001** | 1.3 | 1.1-1.4 |
| Principal psychiatric diagnosis |  |  |  |
| Major depressive disorder | 0.59 | 1.2 | 0.6-2.2 |
| Bipolar disorder | 0.11 | 1.8 | 0.9-3.9 |
| Schizophrenia | 0.32 | 1.5 | 0.7-3.0 |
| Others | — | — | — |
| Medication compliance during the pandemic (poor vs. good) | 0.87 | 1.0 | 0.6-1.8 |
| Bolded values: <0.05; Abbreviations: *CI*: confidence interval; GAD-7: Generalized Anxiety Disorder – 7 items; *OR*: odds ratio. | | | |

Supplementary Table 2. Strengths of depressive symptoms among guardians of hospitalized psychiatric patients

| PHQ-9 items | Strength |
| --- | --- |
| DEP-1: Anhedonia | 0.752 |
| DEP-2: Sad mood | 1.053 |
| DEP-3: Sleep problems | 0.773 |
| DEP-4: Loss of energy | 1.141 |
| DEP-5: Appetite change | 0.918 |
| DEP-6: Guilt feelings | 0.967 |
| DEP-7: Difficulty in concentration | 1.058 |
| DEP-8: Psychomotor signs | 0.944 |
| DEP-9: Suicidal ideation | 0.694 |

Supplementary Table 3. Strengths of depressive symptoms among guardians of hospitalized psychiatric patients after adjusting for anxiety symptoms and fatigue

| PHQ-9 items | Strength |
| --- | --- |
| DEP-1: Anhedonia | 0.732 |
| DEP-2: Sad mood | 0.971 |
| DEP-3: Sleep problems | 0.802 |
| DEP-4: Loss of energy | 1.239 |
| DEP-5: Appetite change | 0.780 |
| DEP-6: Guilt feelings | 0.642 |
| DEP-7: Difficulty in concentration | 0.835 |
| DEP-8: Psychomotor signs | 0.647 |
| DEP-9: Suicidal ideation | 0 |

Supplementary Table 4. Weighted adjacency matrix of the network for global QOL and depressive symptoms among guardians of hospitalized psychiatric patients

|  | DEP-1 | DEP-2 | DEP-3 | DEP-4 | DEP-5 | DEP-6 | DEP-7 | DEP-8 | DEP-9 | Global QOL |
| --- | --- | --- | --- | --- | --- | --- | --- | --- | --- | --- |
| DEP-1 | 0 | 0.415 | 0 | 0.178 | 0 | 0 | 0.119 | 0.026 | 0.002 | -0.008 |
| DEP-2 | 0.415 | 0 | 0.110 | 0.194 | 0.017 | 0.253 | 0 | 0 | 0.084 | -0.030 |
| DEP-3 | 0 | 0.110 | 0 | 0.255 | 0.207 | 0 | 0.028 | 0.134 | 0 | -0.064 |
| DEP-4 | 0.178 | 0.194 | 0.255 | 0 | 0.301 | 0.145 | 0.057 | 0.005 | 0.047 | -0.015 |
| DEP-5 | 0 | 0.017 | 0.207 | 0.301 | 0 | 0.030 | 0.137 | 0.110 | 0.111 | -0.028 |
| DEP-6 | 0 | 0.253 | 0 | 0.145 | 0.030 | 0 | 0.167 | 0.102 | 0.259 | -0.123 |
| DEP-7 | 0.119 | 0 | 0.028 | 0.057 | 0.137 | 0.167 | 0 | 0.400 | 0.158 | -0.089 |
| DEP-8 | 0.026 | 0 | 0.134 | 0.005 | 0.110 | 0.102 | 0.400 | 0 | 0.186 | 0.022 |
| DEP-9 | 0.002 | 0.084 | 0 | 0.047 | 0.111 | 0.259 | 0.158 | 0.186 | 0 | 0 |
| Global QOL | -0.008 | -0.030 | -0.064 | -0.015 | -0.028 | -0.123 | -0.089 | 0.022 | 0 | 0 |

Supplementary Figure 1. Comparison of the network structures for depressive symptoms between the whole sample (N=1,101) and the depressed subgroup (N=357)


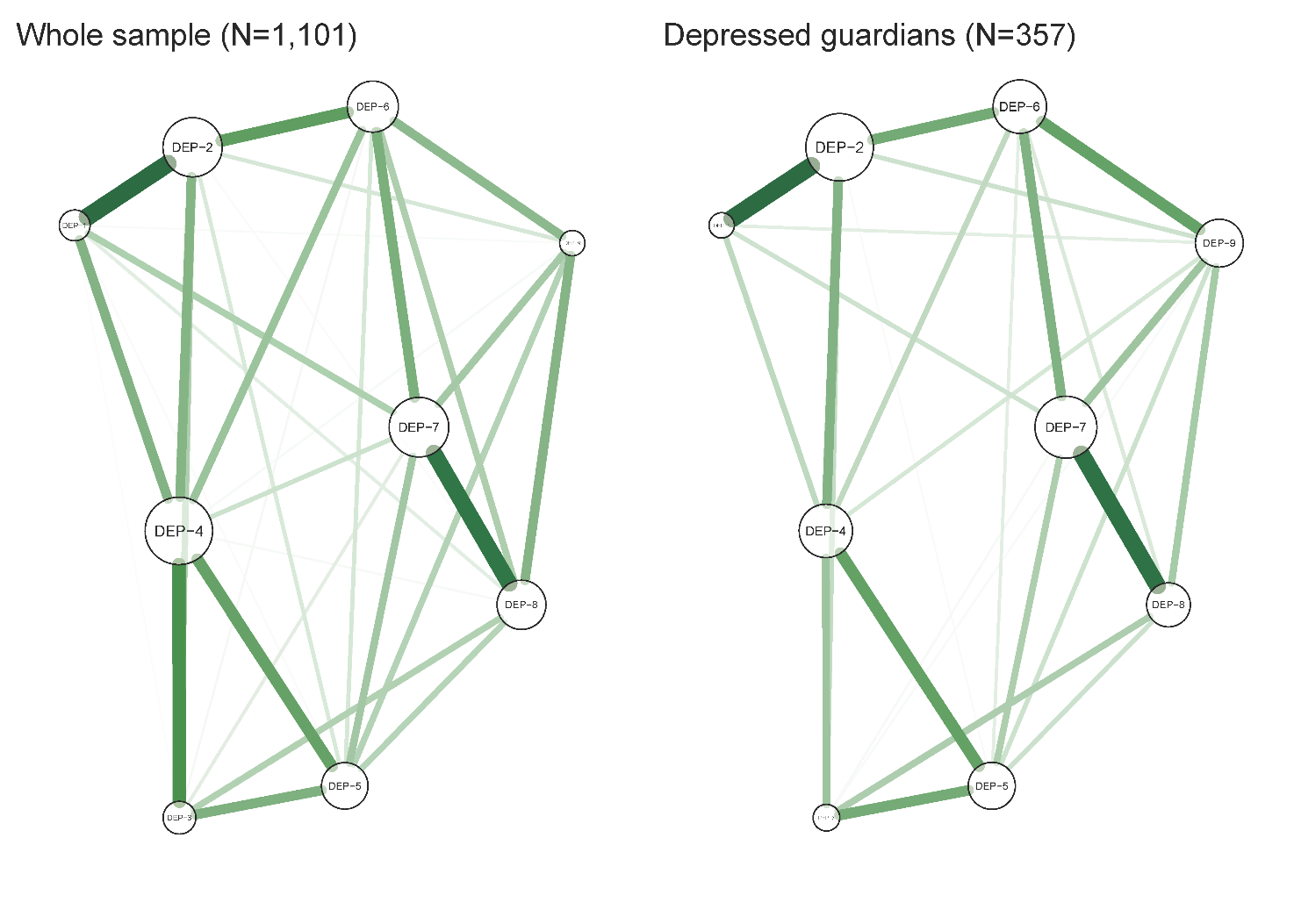


Figure legend: DEP-1: Anhedonia; DEP-2: Sad mood; DEP-3: Sleep problems; DEP-4: Loss of energy; DEP-5: Appetite change; DEP-6: Guilt feelings; DEP-7: Difficulty in concentration; DEP-8: Psychomotor signs; DEP-9: Suicidal ideation.

Supplementary Figure 2. Network invariance test and global strength test of the network structures for depressive symptoms between the whole sample (N=1,101) and the depressed subgroup (N=357)


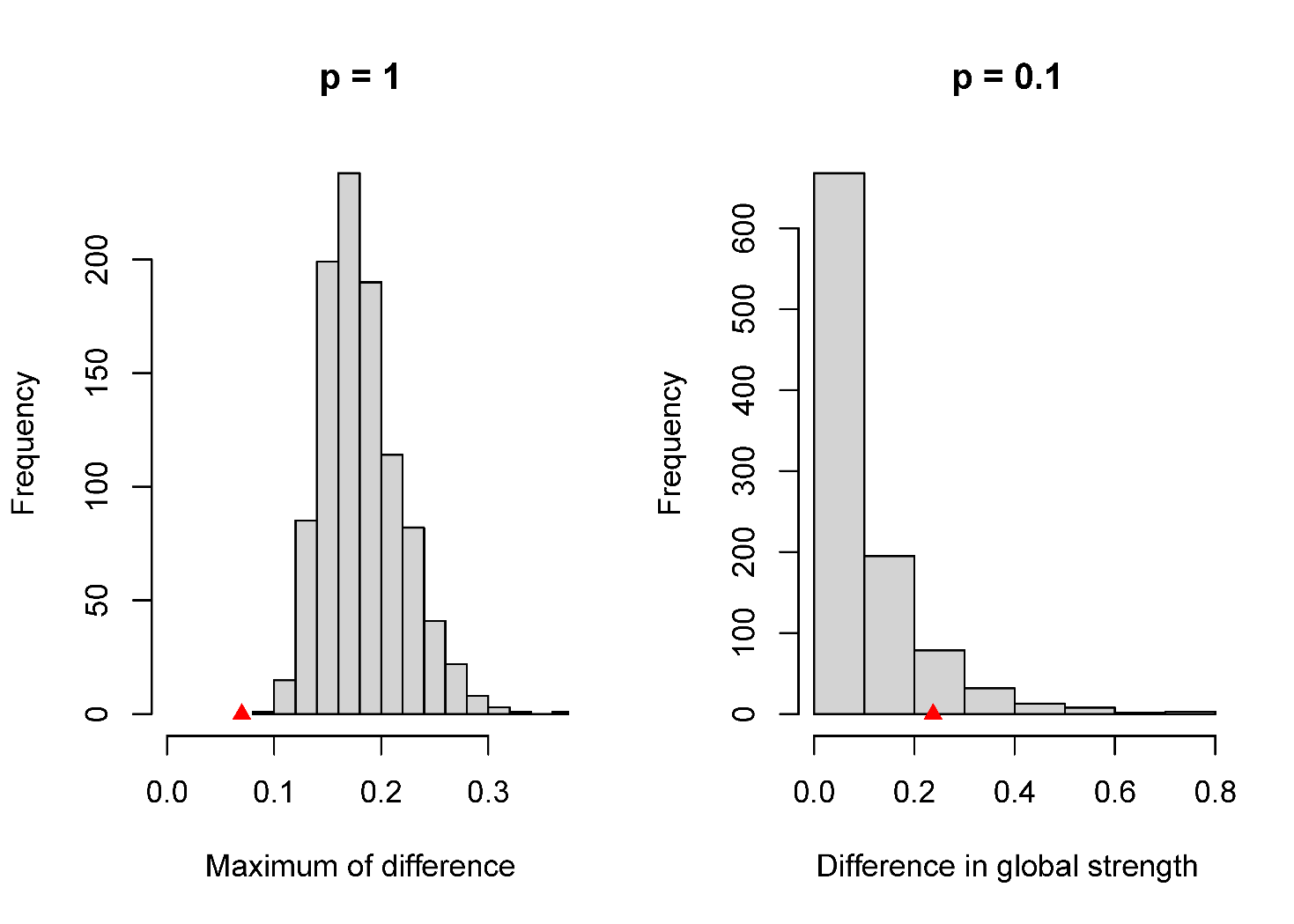


Figure legend: The grey bars indicate the reference distribution under null hypothesis generated from the permutation procedure. The red triangle indicates the observed maximum difference in paired edge weights and the observed difference in global strength between the whole sample and the depressed guardians.

Supplementary Figure 3. Network stability of depressive symptoms among depressed guardians of hospitalized psychiatric patients (N=357)


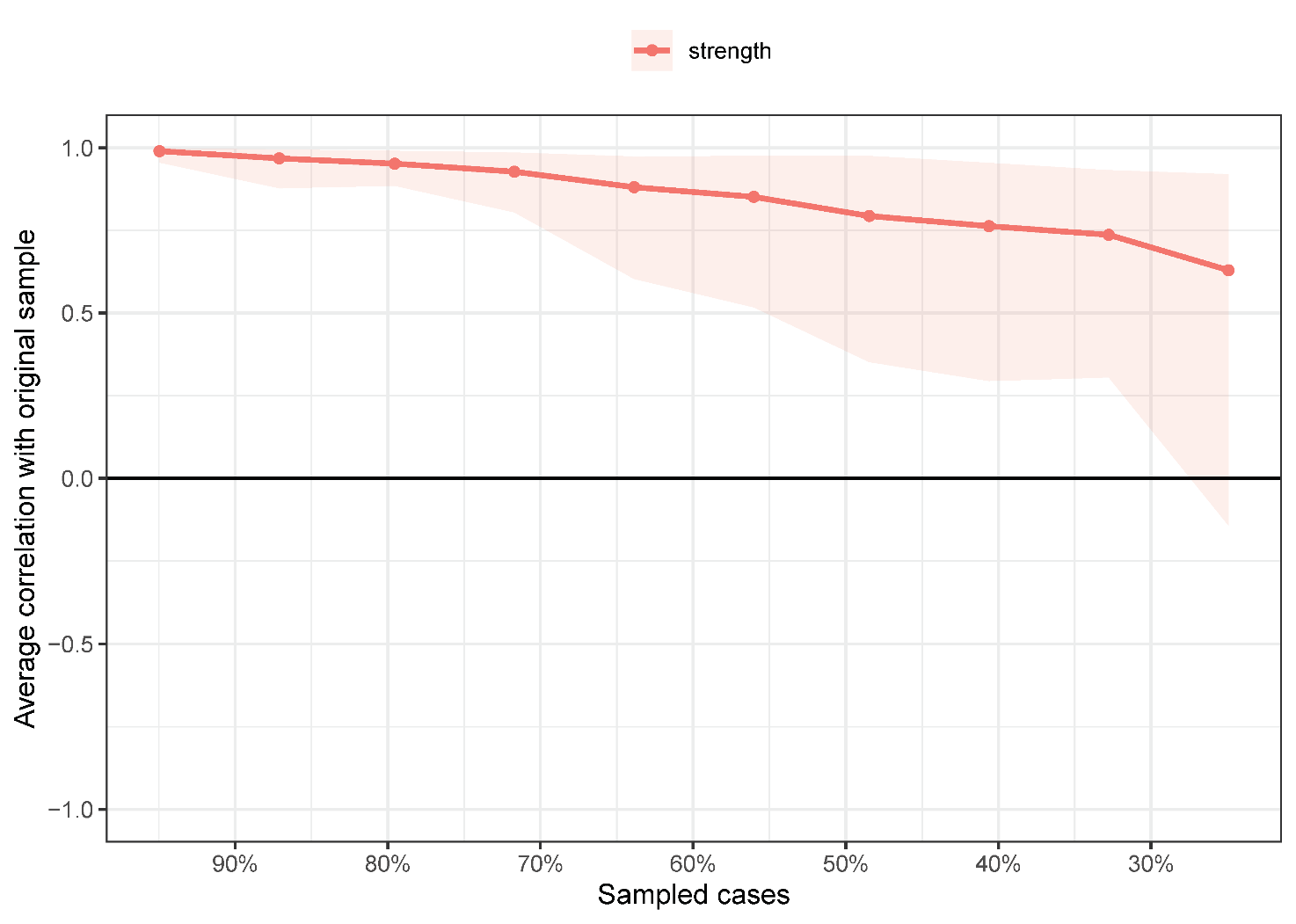


Figure legend: The *CS-C* for network model strength was 0.361, indicating that centrality strength values in the network remained stable after dropping 36.1% of the sample.
